# Antifungal Efficacy of Selected Plant Essential Oils Against Clinical Canine Isolates *Malassezia pachydermatis*

**DOI:** 10.3390/microorganisms13122675

**Published:** 2025-11-24

**Authors:** Eva Čonková, Peter Váczi, Zuzana Malinovská

**Affiliations:** Department of Pharmacology and Toxicology, University of Veterinary Medicine and Pharmacy, 041 81 Košice, Slovakiazuzana.malinovska@uvlf.sk (Z.M.)

**Keywords:** *Malassezia pachydermatis*, essential oils, antifungal efficacy, minimum inhibitory concentration

## Abstract

*Malassezia pachydermatis*, an important opportunistic secondary pathogen, is often associated with atopic dermatitis or otitis externa in dogs. Recent studies indicate an increase in resistance of this yeast to commonly used antifungal agents. Therefore, the search for new antifungal agents is a challenge. In the present study, the susceptibility of *M. pachydermatis* strains to 10 plant essential oils—EOs (bergamot, grapefruit, coriander, hyssop, lavender, tea tree, nutmeg, oregano, rosemary, and Spanish sage) was determined using the broth microdilution method. All 15 clinical strains tested were susceptible to coriander (100%). A good antifungal activity was shown for EO from nutmeg (93.33%), bergamot (86.66), Spanish sage and hyssop (73.33%) and rosemary (66.67%). Lower antifungal efficacy was identified in EOs from grapefruit, lavender, tea tree and oregano (53.33%). The obtained results indicate promising prospects for the clinical use of essential oils in the treatment of *M. pachydermatis* infections.

## 1. Introduction

*Malassezia pachydermatis* is considered an important opportunistic secondary pathogen, often detected in atopic dermatitis or otitis externa in dogs. The onset of *Malassezia* infection depends on the host immune system and expression of virulence factors such as enzymes (esterase, lipase, lipoxygenase, protease, chondroitin sulphatase, hyaluronidase and phospholipase) that play an important role in the colonisation and multiplication of the yeast and trigger the release of inflammatory mediators [1,2].

Treatment of *Malassezia* infections mostly includes the application of topical formulations containing antifungal drugs, especially polyenes (nystatin) and azoles (clotrimazole, itraconazole, fluconazole, posaconazole, and miconazole), often in combination with anti-inflammatory agents, antibiotics and chlorhexidine [3,4,5]. Recent studies have revealed an increase in the resistance of fungal strains to commercially available antifungal agents, suggesting that the development of new antifungals is an essential strategy to overcome problems encountered in the treatment of these infections [6,7].

Plant essential oils, known for their medicinal properties (analgesic, sedative, anti-inflammatory, anti-spasmodic, local anaesthetic, and anticarcinogenic) and due to their antimicrobial activity, appear to be a relevant alternative [8,9,10]. Essential oils are mixtures of secondary metabolites of many aromatic plants containing a variety of volatile compounds, including terpenes, aldehydes, alcohols, ketones and simple phenolics whose fungal properties have been proven [11,12]. The antifungal activity of EOs has not yet been fully elucidated. Since EOs represent a highly complex chemical composition (approximately 20–60 components at different concentrations), their activity may be related to different strategies by targeting crucial components within fungal cells. Similarly to the conventional antifungal drugs, EOs can directly bind to ergosterol, an essential sterol component in fungal cell membranes, or modulate its production, disrupting membrane integrity, which results in cell death. Some EOs prevent cell wall formation by targeting beta-glucans, which weakens the cell structural framework and leads to cell destruction. Another mode of action of EOs is the inhibition of the activity of mitochondrial dehydrogenases in the tricarboxylic acid cycle (e.g., malate dehydrogenase, succinate dehydrogenase), enzymes responsible for energy production in mitochondria. Disruption of the mitochondrial electron transport chain reduces membrane potential and ATP levels, induces excess reactive oxygen species (ROS) production accompanied with the release of cytochrome c from mitochondria into cytosol leading to indirect induction of the cell apoptosis. In addition, EOs are able to inhibit efflux pumps, which expel drugs from the cell, and allow drugs to accumulate within the fungal cell, improving efficacy and potentially reducing the drug dosage [8,13,14,15].

The literature provides a wealth of information on the antifungal activity of EOs against *Candida* species, but only a few studies address the effectiveness of EOs against *M. pachydermatis*. The aim of this study was therefore to determine the susceptibility of *M. pachydermatis* strains to ten plant EOs (bergamot, grapefruit, coriander, hyssop, lavender, tea tree, nutmeg, oregano, rosemary, and Spanish sage), which are rarely tested by the microdilution method, and to investigate their potential use for the treatment of *Malassezia* infections.

## 2. Material and Methods

### 2.1. Malassezia pachydermatis Strains

A total 15 clinical isolates from the ear swabs of dogs without current manifestation and recent history of otitis externa were used for testing. Samples obtained from the Small Animal Clinic of the University of Veterinary Medicine and Pharmacy in Košice (Slovakia) were taken from dogs of different breeds, sexes and ages. All strains used for the experiments were identified and confirmed based on their phenotypic and genotypic characteristics described by Kaneko et al. [16] and Gaitanis et al. [17]. Until the beginning of the experiments, the strains were preserved at −80 °C in freezing medium (100 µL 60% gly cerol and 300 µL medium—glucose 4 g, tryptophan 1 g, yeast extract 0.5 g per 100 mL). Before use, the yeasts were subcultured twice on SAOT (Sabouraud’s dextrose agar—SDA supplemented with glycerol—2 mL, Tween 80—2 mL, Tween 40—5 mL and olive oil—5 mL per litre) and incubated at 35 °C for 96 h.

The reference strain of *M. pachydermatis* CBS 1879 (Centraalbureau voor Schimmelcultures, Utrecht, The Netherlands) was also included in the study.

### 2.2. Plant Essential Oils Tested

The antifungal activity of ten plant essential oils (Table 1): bergamot—*Citrus aurantium* L. subsp. *bergamia*, grapefruit—*Citrus paradisi*, coriander—*Coriandrum sativum* L., hyssop—*Hyssopus officinalis* L., lavender—*Lavandula angustifolia* MILLER., tea tree—*Melaleuca alternifolia*, nutmeg—*Myristica fragrans* Houtt, oregano—*Origanum vulgare* L., rosemary—*Rosmarinus officinalis* L., and Spanish sage—*Salvia lavandulifolia* Vahl (Calendula a. s., Nová Ľubovňa, SR) was investigated.

### 2.3. Testing Quality Control

Testing quality control was verified by determining the susceptibility of the reference strain *Candida parapsilosis* ATCC 22019 (Czech Collection of Microorganisms, Brno, Czech Republic) to itraconazole (Sigma Aldrich, St. Louis, MO, USA).

### 2.4. Determination of Minimum Inhibitory Concentration (MIC) of Tested Antifungal Agents

To determine the susceptibility of *M. pachydermatis* strains, the standard microdilution method M 27-A3 [18] was used with slight modifications.

The test procedure consisted of the preparation of yeast inoculum and concentrations of tested agents. *M. pachydermatis* strains were first suspended in sterile PBS (Phosphate-Buffered Saline) solution containing 0.1% Tween 80 to an optical density at McFarland 1, yielding 1–5 × 10^6^ CFU/mL (Colony Forming Units/mL). This suspension was further diluted with SBOT (Sabouraud’s broth medium supplemented with the same substances as SAOT) to 10^4^ CFU/mL. As for *C. parapsilosis* ATTC 22019, the inoculum was prepared from 24 h-old yeast cultured on SDA—Sabourad’s dextrose agar (HiMedia Laboratories Pvt. Ltd., Mumbai, India) and suspended in sterile saline solution to an optical density at McFarland 0.5, resulting in 1–5 × 10^6^ CFU/mL, and subsequently diluted to 10^3^ CFU/mL in SBG (Sabouraud dextrose broth; Hi Media Laboratories Pvt. Ltd., Mumbai, India; supplemented with 10 mM glucose).

The stock solutions of EOs were prepared at a concentration of 400,000 μg/mL by dissolving 100% EOs in sterile paraffin oil and diluted by binary dilution using SBOT to the concentrations of 400,000–800 μg/mL directly in 96-well microplates.

A stock solution of itraconazole (1600 µg/mL), a water insoluble antifungal agent, was prepared by dissolving the powdered substance in DMSO (dimethyl sulfoxide, Sigma Aldrich, Schnelldorf, Germany) and used to prepare a series of two-fold dilutions ranging from 32 to 0.0625 μg/mL, with SBG in microplates.

In both cases, microplate wells 1–10 contained descending concentrations of test agents (100 μL) and inoculum (100 μL). This resulted in the halving of the concentration of the tested agents, for EOs ranging from 200,000 to 400 μg/mL and for itraconazole from 16 to 0.0313 μg/mL. Well 11 served as a negative control containing 200 μL of SBOT or SBG. Well 12 (positive control) contained 100 μL of SBOT or SBG and 100 μL of inoculum. Microplates inoculated with *M. pachydermatis* samples were incubated at 35 °C for 72 h and those with *Candida parapsilosis* ATCC 22019 were incubated at 35 °C for 24 h. After incubation, the minimum inhibitory concentration (MIC) was read.

To better evaluate the MIC (minimal inhibitory concentration) end-points, 10 µL of 0.1% resazurin (sterilised through 0.22 µm filter) was added into each well of microplate six hours before reading the results. Inhibition of yeast growth was determined at the MIC that prevented the colour shift from blue (no yeast growth) to orange-pink (yeast growth) [19].

Since there are no criteria for assessing the susceptibility of *M. pachydermatis* to EOs, the tested strains were classified according to the following criteria: susceptible (S)—MIC sample ≤ MIC50; susceptible dose-dependent (S-DD)—MIC50 < MIC sample ≤ MIC90 and resistant (R)—MIC sample > MIC90 [20].

The susceptibility of *C. parapsilosis* ATCC 22019 to itraconazole was evaluated according to the criteria established in the M-27-A3 method (S—≤0.125 μg/mL, S-DD—0.25–0.5 μg/mL and R ≥ 1 μg/mL). The average MIC value from the three measurements was 0.0313 µg/mL, which is in accordance with the limit set out in the methodology.

### 2.5. Statistical Analysis

The MIC values were determined based on repeating the experiments twice and average values were taken. The data are presented as average ($\bar{x}$), standard deviations (SD), mode and median. The analysis of the mean MIC values of the plant essential oils tested was performed using one-way ANOVA followed by Tukey’s multiple comparison test (GraphPad Prism 8.0.1, San Diego, CA, USA). The level of statistical significance was set up at *p* ˂ 0.05.

## 3. Results

Table 2 summarises the statistical analyses of the achieved MIC in EOs tested. The lowest MIC was identified for EO from coriander, with an average MIC of 400 µg/mL, followed by EO from lavender (MIC 586.67 µg/mL), hyssop (MIC 666.67 µg/mL), rosemary (MIC 741.67 µg/mL), Spanish sage (MIC 816.67 µg/mL), bergamot (MIC 821.67 µg/mL), oregano (MIC 2430 µg/mL) and tea tree (MIC 2360 µg/mL). Higher MIC values were noticed for EOs from nutmeg (MIC 5838.33 µg/mL) and grapefruit (MIC 15,015 µg/mL). Statistically significant differences (*p* ˂ 0.05) when comparing the tested EOs with each other were observed between grapefruit EO and the EOs of bergamot, coriander, hyssop, lavender, rosemary and Spanish sage.

**Figure 1:**
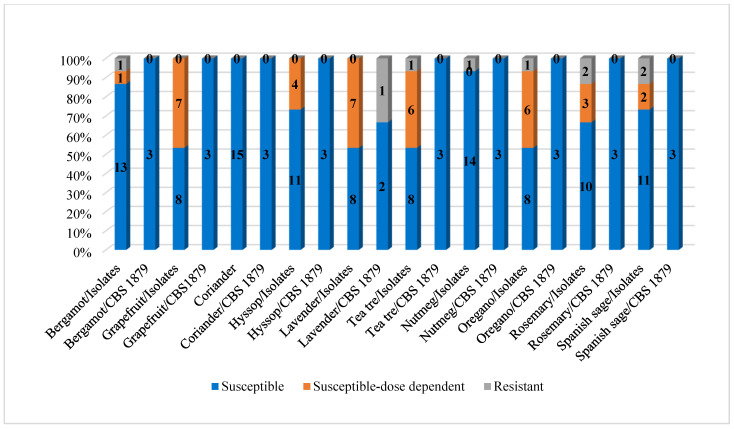
Susceptibility of *M. pachydermatis* strains to the tested plant EOs.

Table 3 and Figure 1 illustrate the susceptibility of *M. pachydermatis* clinical isolates and reference strain to the tested plant EOs.

All 15 clinical strains were susceptible to coriander (100%). Up to 14 isolates were susceptible to nutmeg (93.33%) and 13 isolates (86.66%) to bergamot. EOs from hyssop and Spanish sage showed antifungal activity against 11 isolates (73.33%) and EO from rosemary against 10 isolates (66.67%). Eight strains (53.33%) were susceptible to grapefruit, lavender, tea tree and oregano EOs. Resistance of *M. pachydermatis* clinical strains was found in only one isolate (6.67%) to EOs from bergamot, tea tree, nutmeg and oregano, and in two isolates (13.33%) to EOs from rosemary and Spanish sage. Susceptibility of the *M. pachydermatis* CBS 1879 strain was detected in all tested EOs, except for lavender, where two strains (66.67%) exhibited susceptibility and one strain (33.33%) was resistant.

## 4. Discussion

The significant anti-*Malassezia* effectiveness of EOs has been reported in several studies in human medicine, but less so in the field of veterinary medicine [3,6,7,21,22]. Plant EOs, as a natural product of a specific chemical composition, usually contain two or three major components, mostly terpenes, at a relatively high concentration (20–90%), while other components (aromatic and aliphatic compounds) are less abundant. These components are responsible for their diverse biological activities that arise from their synergistic interaction [23]. The anti-*Malassezia* activity of EOs varies not only depending on the type of EOs used, but also on the *Malassezia* species tested. As for *M. pachydermatis*, Bismark et al. [22] tested 22 EOs against 15 *M. pachydermatis* isolates using the agar disc diffusion method and reported on a strong antifungal effect of EOs from lemon grass, cinnamon leaf, clove, manuka, Indian melissa, oregano, palmarosa, and winter savoury. In another study, it was found that out of six EOs (*Artemisia sieber*, *Heracleum persicum*, *Menta spicata*, *Rosmarinus officinalis*, *Thymus kotschyanus*, and *Zataria multiflora*) examined for anti-*M. pachydermatis* activity, *Z. multiflora* (60 µg/mL) and *A. sieberi* (80 µg/mL) exhibited the highest inhibitory effects [6].

In the present study, the antifungal activity of ten EOs was evaluated, the efficacy of which against *M. pachydermatis* was tested by the microdilution method is rarely reported in the literature. The best effectiveness was found for coriander. The effect of coriander EO extracted from the seeds against *M. pachydermatis* was tested by Bismarck et al. [22] using a disc diffusion test. The 20% (200,000 µg/mL) solution gave the inhibition zone ranging from 19 to 25 mm. Good antifungal activity of the EO from *C. sativum* was also demonstrated against *C. parapsilosis* CBS 604 (125 µg/mL), *C. dubliniensis* CBS 7987 and *C. krusei* CBS 573 (250 µg/mL) and *C. albicans* CBS 562 (500 µg/mL) [24]. Coriander seed oil is known for its broad spectrum of antimicrobial (antibacterial and antifungal) and pharmacological activities (antiedemic, anti-inflammatory, antiseptic, antidiabetic, emmenagogue, antihypertensive, lipolytic and myorelaxant) [25]. Laribi et al. [26] analysed the EO composition of *C. sativum* from different origins and found that linalool as the principal component that may exert antifungal activity ranged from 37.65% from Bangladesh to 87.54% from Tunisia. As the authors state, differences in the chemical profile of coriander EOs across regions may be influenced by the genetic, climatic, seasonal and geographic conditions. The results of our study are similar to the findings of Begnami et al. [24], as the coriander EO MIC reached 400 µg/mL with a linalool content of 64 ± 2%.

Lavender, hyssop, rosemary, Spanish sage and bergamot also demonstrated remarkable antifungal efficacy against *M. pachydermatis* with MIC values ranging from 586.67 µg/mL to 821.67 µg/mL.

Lavender EOs or extracts are known for their broad spectrum of pharmacological activities, allowing for their use as sedatives, anti-inflammatories, antioxidants, antimicrobials, antifungals, insecticides, and larvicide. The chemical composition of lavender EOs is mainly characterised by the presence of oxygenated monoterpenes (e.g., linalool, linalyl acetate, 1,8-cineole, camphor) and irregular monoterpenoids (e.g., lavandulol and lavandulyl acetate) [27]. In an experiment described by Váczi et al. [21] using a disc diffusion method, lavender EO inhibited the growth of *M. pachydermatis* at 30% (300,000 µg/mL) concentration, while no yeast susceptibility was recorded at a concentration of 5% (50,000 µg/mL) and 0.5% (5000 µg/mL). However, in the present study the strains exhibited the susceptibility at a concentration of 586.67 µg/mL (0.057%) which may be influenced by the different susceptibility of the tested isolates.

In traditional medicine, *Hyssopus* spp. are used for their curative properties against cough, cold, loss of appetite, fungal infection, and spasmodic conditions. Essential oil constituents of hyssop exhibited antimicrobial, antifungal, and muscle relaxant properties. The major components of hyssop are monoterpene pinocamphone, isopinocamphone, and β-pinene [28]. Hristova et al. [29] evaluated the antifungal activity of hyssop EO against clinical isolates and reference strains of the genus *Candida*. The highest susceptibility was shown by *C. albicans* strains with MIC of 210.3 ± 62.3 µg/mL, followed by *C. krusei* (MIC = 224.0 ± 64.0 µg/mL), *C. parapsilosis* (MIC = 298.7 ± 104.5 µg/mL), *C. tropicalis* (MIC = 682.7 ± 264.4 µg/mL) and *C. glabrata* (MIC = 768.0 ± 280.4 µg/mL). The authors also tested the influence of the chemical composition of hyssop oil on its anticandidal activity. Out of the major EO constituents and their isomers, such as cis-pinocamphone (48.98–50.77%), β-pinene (13.38–13.54%), trans-pinocamphone (5.78–5.94%) and β-phellandrene (4.44–5.17%), cis- and trans-pinocamphone were the most active constituents with MIC values 28% and 21% higher than those of hyssop oil against *C. albicans* and *C. glabrata*, respectively.

Rosemary EO has gained considerable scientific interest due to its wide range of pharmacological activities (anticancer, anti-inflammatory, and antioxidant). It has also been shown to promote healing, angiogenesis, and improvements in granulation tissue, it accelerates wound healing, and enhances the survival and viability of tissues while reducing tissue necrosis. In addition, rosemary EO exhibits antifungal properties. In traditional medicine, it is used to relieve dysmenorrhea, renal colic pain, and respiratory disorders due to its antispasmodic properties [30]. The antifungal activity of rosemary EO was tested by Waller et al. [31] against pathogenic strains of *M. pachydermatis* isolated from canine and feline otitis. The MIC ranged from ≤0.078 to >2.5 mg/mL (78–2500 µg/mL), while in the present study it ranged from 400 µg/mL to 3125 µg/mL. Among all 19 compounds identified in rosemary EOs by Waller et al. [31], 1,8-cineole (11–49.4%), camphor (16–17.8%) and α-pinene (2–12.2%) prevailed, whereas in the EO tested in our study, cineole was present at 25 ± 1%, followed by α-pinene and camphor (19 ± 1%). Similarly, Khoshravi et al. [6] reported the MIC_90_ of 360 µg/mL for the inhibitory effect of rosemary EO against *M. pachydermatis*—unfortunately without providing information on the presence of the major components that may play an important role in the antifungal activity.

*Salvia lavandulifolia* Vahl is a valuable aromatic and medicinal plant with a high economic potential in agriculture, cosmetics and pharmacology. The medicinal activities are attributed to its essential oils and include anti-spasmodic, antiseptic, analgesic, oestrogenic, anticholinesterase, antioxidant, anti-inflammatory, and central nervous system-suppressing activities, as well as antimicrobial effects. The predominant constituents in the Spanish sage EO are monoterpenes such as α- and β-pinene, 1,8-cineole, and camphor, the proportions of which may be significantly affected by various environmental and genetic factors, as well as by phenological stages (vegetative and full flowering stages) [32,33,34]. Pisteli et al. [3] tested the anti-*M. pachydermatis* efficacy of EO from *Salvia sclarea* with no inhibition of the yeast growth (MIC => 10%; 100,000 µg/mL). The major constituent of the EO were linalool (28.4%) and linalyl acetate (48.9%), while in the EO from *S. lavandulifolia* Vahl examined in this study, camphor represented the main component (27 ± 1%), and the mean MIC reached 816.67 µg/mL.

Bergamot EO has not only been reported for its antibacterial and antifungal activity, but it is also known for its anti-inflammatory, antiproliferative, neuro-pharmacological and neuroprotective (calming, relaxing, anxiolytic), and analgesic activities. In general, the volatile fraction with dominated monoterpene hydrocarbons such as limonene (25–55%), β-pinene (4–11%), and γ-terpinene (5–11%) is considered to be a bearer of these pharmacological properties [35]. However, compared to other *Citrus* EOs, bergamot EO is characterised by a much lower percentage of limonene and allied monoterpenes and a much higher content of oxygenated terpenes, specifically linalool (2–20%) and linalyl acetate (15–10%) [36]. In the study by Lee and Lee [37], no efficacy of bergamot EO was found against *M. pachydermatis*, while Váczi et al. [21] noticed a small inhibition zone (8.44 ± 1.67 mm) for bergamot EO at a concentration of 30% (300,000 µg/mL). Despite these findings, this study proved the efficacy of bergamot EO with an average MIC of 821.67 µg/mL, which is consistent with the study by Limtaniakul et al. [38] confirming the anti-*Malassezia* effect of bergamot EO.

Higher MIC values were required for EO from oregano, tea tree, nutmeg and grapefruit (mean MIC 2360–15,015 µg/mL) to achieve sufficient efficacy against *M. pachydermatis* strains used in this experiment.

Tea tree oil has been found to be effective in treating small superficial wounds and insect bites, treating small ulcers (furuncles and mild acne), relieving itching and irritation in mild athlete’s foot, and in the symptomatic treatment of moderate oral mucosal inflammation [39]. Several research articles point to the anti-*M. pachydermatis* effect of tea tree EO [21,22,40,41]. For instance, Weseler et al. [40] observed the antifungal activity against clinical isolates *M. pachydermatis* in the MIC range of 560–1120 µg/mL. Tea tree EO consisted mainly of terpinene-4-ol (40.7%), γ-terpinene (20.4%), α-terpinene (9.1%), p-cymene (2.2%), 1,8-cineol (3.1%), terpinolene (3.3%), α-terpineol (3.1%), α-pinene (2.3%), and limonene (1.0%). The antifungal activity of tea tree EO, performed using the disc diffusion method in the experiment by Lee and Lee [37], showed a strong inhibitory effect on *M. pachydermatis* at a concentration of 2 mg/mL (2000 µg/mL) with an inhibition zone of 1.5 cm. The major constituents of the EO were γ-terpinolene (17.96%) and terpinen-4-ol (45.54%). Similar results were found in this study, where the average MIC achieved 2360 µg/mL and the main component of the tested EO, terpinen-4-ol, was 33 ± 2%.

Oregano EO has been shown to possess several bioactive properties including antioxidant, antimicrobial (antifungal, bactericidal and antiviral), anti-inflammatory, analgesic, antihypertensive, antiproliferative, and antidiabetic [42,43]. Pisteli et al. [3] and Waller et al. [31] report on the anti-*Malassezia* activity of *Origanum vulgare* L. EO. In the study by Waller et al. [31], the inhibitory and fungicidal activity (MIC_90_ and MFC_90_) of oregano EO investigated against *M. pachydermatis* isolates collected from dogs suffering from otitis achieved a concentration of 0.625 mg/mL (625 µg/mL) for both. Among all 16 identified compounds, carvacrol (73.9%) was the major component observed in oregano oil. The lower antifungal activity of oregano EO tested in our study (average MIC = 2430 µg/mL) may be related to the lower amount of carvacrol (57 ± 3%).

As for nutmeg EO, clinical and experimental investigations have confirmed the antioxidant, antimicrobial, anti-inflammatory, anticancer, antimalarial, anticonvulsant, hepatoprotective, antiparasitic, insecticidal, and nematocidal activities [44]. However, no articles were found focusing on the antifungal activity of EO from *Myristica fragrans* (nutmeg) against *M. pachydermatis*. Nicolic et al. [45] present the antifungal activity of nutmeg EO against the *C. albicans* ATCC 2091 strain. Using the disc diffusion method, the growth inhibition zone of 28 mm was noticed. The efficacy was attributed to the monoterpene hydrocarbon sabinene (42.3%), which was identified in the most abundant amount among the twenty-five compounds. Nutmeg EO is also characterised by the presence of other monoterpenes such as β-pinene (26%), α-pinene (10.51%) and γ-terpinen (8.51%) [46] and aromatic compounds such as myristicin (1.8 to 12.8%) and elemicin (4.3 to 11.1%) [47]. The composition of nutmeg EO tested in our study corresponds to these studies, although a higher MIC was required for sufficient anti-malassezia efficacy (mean MIC = 5838.33 µg/mL).

Unlike other EOs, EO from *Citrus paradisi* (grapefruit) demonstrated the anti-*M. pachydermatis* activity at the highest MIC (average MIC = 15,015 µg/mL). The obtained results are close to the study by Pisteli et al. [3], who observed the antifungal activity against *M. pachydermatis* at MIC and MFC of 1.3% (13,000 µg/mL) for both. The content of the main constituent in the tested oil was limonene (91.7%), like in the tested grapefruit EO in the present study (87 ± 3%). According to the available literature, the grapefruit EO possesses antiseptic, healing, soothing, curing and rubifacial qualities, as well as antimicrobial activity [48].

Although the present study has some limitations (no standardised method for testing the susceptibility of *M. pachydermatis* to antifungals and no criteria for assessing the susceptibility are established), it can be considered a basis for future studies of the efficacy of EOs in vivo. Significant antifungal efficacy of tested plant EOs was found in vitro with different MIC values (the lowest MIC for coriander EO—400 µg/mL and the highest MIC for grapefruit EO—15,015 µg/mL) which may be affected by the chemical composition of EOs. Moreover, the obtained results point to the antifungal activity of plant EOs, whose anti-*M. pachydermatis* activities have either not been studied so far (nutmeg) or have been rarely evaluated using the microdilution method. Based on the accessible articles, we can confirm that the efficacy of EOs depends not only on the amount of the major components, but may also be influenced by the virulence of the individual *M. pachydermatis* strain, resulting in different susceptibility [22].

## 5. Conclusions

The results obtained in this study demonstrate that plant EOs appear to be a promising alternative for the treatment of infections caused by *M. pachydermatis*. However, their implementation in clinical practice as effective natural agents or adjuncts to conventional antifungal drugs, as well as their safe use in veterinary dermatology, require further research, with particular emphasis on assessing their cytotoxicity.

## Figures and Tables

**Table 1:** Characteristics of essential oils used in the experiment *.

Essential Oil	Botanical Name	Family	Plant Part	Major Components
Coriander	*Coriandrum sativum* L.	*Apiaceae*	Fruit	Linalool (64 ± 2%)
Hyssop	*Hyssopus officinalis* L.	*Lamiaceae*	Aerial part of the plant	Pinocamphone (50.0 ± 2%) Isopinocamphone (28.0 ± 1%) α-pinene (11.0 ± 1%)
Lavender	*Lavandula angustifolia* MILLER		Flower	Linalool (48 ± 2%)
Rosemary	*Rosmarinus officinalis* L.		Leaf	1,8-cineole (25.0 ± 1%) α-pinene (19.0 ± 1%) Camphor (19.0 ± 1%)
Spanish sage	*Salvia lavandulifolia* Vahl		Aerial part of the plant	Camphor (27 ± 1)
Oregano	*Origanum vulgare* L.		Aerial part of the plant	Carvacrol (57 ± 3%)
Tea tree	*Melaleuca alternifolia* Cheef	*Myrtaceae*	Leaf	Terpinen-4-ol (33 ± 2)
Nutmeg	*Myristica fragrans* Houtt	*Myristicaceae*	Core	α-pinene (18.0 ± 1%) Sabinene (14.0 ± 1%) β-pinene (13.0 ± 1%) Myristicin (5 ± 0.2%)
Bergamot	*Citrus aurantium* L. subsp. *bergamia*	*Rutaceae*	Pericarp	Limonene (36 ± 1%) Linalyl acetate (23 ± 1%) Linalool (15 ± 1%)
Grapefruit	*Citrus paradisi* Macfad		Pericarp	Limonene (87 ± 3%)

* Note—The table was prepared based on certificates obtained from the company Calendula a. s. (Nová Ľubovňa, SR). The major components were identified using gas chromatography analyses.

**Table 2:** Evaluation of plant essential oil MIC (µg/mL).

Parameter	Bergamot	Grapefruit	Coriander	Hyssop	Lavender	Tea Tree	Nutmeg	Oregano	Rosemary	Sage
Clinical isolates										
Minimum	400	800	400	400	400	400	1600	400	400	400
Maximum	3125	100,000	400	1600	800	25,000	12,500	12,500	3125	3125
$\bar{x}$	821.67 ^a^	15,015 ^a,b,c,d,e,f^	400 ^b^	666.67 ^c^	586.67 ^d^	2360	5838.33	2430	741.67 ^e^	816.67 ^f^
SD	715.07	34,516.95	0	493.77	206.56	6281.13	2528.85	3057.90	736.53	947.44
Mode	400	3125	400	400	400	400	6250	3125	400	400
Median	800	1600	400	400	400	400	6250	1600	400	400
MIC50	800	1600	400	400	400	400	6250	1600	400	400
MIC90	1600	100,000	400	1600	800	1600	6250	3125	800	800
*Malassezia pachydermatis* CBS 1879										
Minimum	800	1600	400	400	400	400	1600	400	400	400
Maximum	800	1600	400	400	1600	400	3125	1600	400	400
$\bar{x}$	800 ^h^	1600	400 ^g,^	400 ^i^	800 ^l^	400 ^j^	2616.67 ^g,h,i,j,k,l,m,n^	1200 ^k^	400 ^m^	400 ^n^
SD	0	0	0	0	692.82	0	880.46	692.82	0	0
Mode	800	1600	400	400	400	400	3125	1600	400	400
Median	800	1600	400	400	400	400	3125	1600	400	400

$\bar{x}$—average of MIC; MIC_50_ and MIC_90_—minimal inhibitory concentration at which 50% or 90% of the strains were inhibited; SD—standard deviation, ^a–n^—MIC values with the same superscript letter are statistically significantly different (*p* ˂ 0.05).

**Table 3:** Susceptibility of *Malassezia pachydermatis* strains to tested plant essential oils.

Parameter	Bergamot	Grapefruit	Coriander	Hyssop	Lavender	Tea Tree	Nutmeg	Oregano	Rosemary	Sage
Clinical isolates										
S (n/%)	13/86.66	8/53.33	15/100	11/73.33	8/53.33	8/53.33	14/93.33	8/53.33	10/66.67	11/73.34
S-DD (n/%)	1/6.67	7/46.67	0	4/26.67	7/46.67	6/40	0	6/40	3/20	2/13.33
R (n/%)	1/6.67	0	0	0	0	1/6.67	1/6.67	1/6.67	2/13.33	2/13.33
*Malassezia pachydermatis* CBS 1879										
S-DD (n*/%)	3/100	3/100	3/100	3/100	2/66.67	3/100	3/100	3/100	3/100	3/100
Resistant (n*/%)	0	0	0	0	1/33.33	0	0	0	0	0

S—susceptible, S-DD—susceptible dose-dependent, R—resistant, n—number of strains, n*—the reference strain was tested in triplicate.

## Data Availability

The original contributions presented in this study are included in the article. Further inquiries can be directed to the corresponding author.
